# RETRACTION: Sundarban Honey Confers Protection against Isoproterenol‐Induced Myocardial Infarction in Wistar Rats

**DOI:** 10.1155/bmri/9787512

**Published:** 2026-08-12

**Authors:** BioMed Research International

**RETRACTION**: R. Afroz, E. M. Tanvir, N. Karim, M. S. Hossain, N. Alam, S. H. Gan, M. I. Khalil, “Sundarban Honey Confers Protection against Isoproterenol‐Induced Myocardial Infarction in Wistar Rats,” *BioMed Research International*, 2016, 6437641, 10.1155/2016/6437641

The above article, published online on 17 May 2016 in Wiley Online Library (onlinelibrary.wiley.com), has been retracted by agreement between the journal Editor‐in‐Chief, Dr Yoel Klug; and John Wiley & Sons Ltd. The retraction has been agreed due to multiple concerns identified in Figure 5, some of which are raised on PubPeer [1]. Further investigation identified additional concerns with the Figure, specifically:

● Panel D appears identical to panel C in Figure 7 of [2]
● Panel C appears identical to panel D in Figure 7 of [2]
● Panel D appears identical to panel C in Figure 3 of [3]
● Panel A appears identical to panel A in Figure 4 of [4]
● Panel B appears identical to panel B in Figure 4 of [4]

Following an assessment of the concerns raised, together with the author’s response and the absence of original image data, the results and conclusions can no longer be considered reliable. The article is therefore retracted.

The authors agree with the retraction.
